# Using Fuzzy Logic to Increase Accuracy in Mango Maturity Index Classification: Approach for Developing a Portable Near-Infrared Spectroscopy Device

**DOI:** 10.3390/s22249704

**Published:** 2022-12-11

**Authors:** Ali Khumaidi, Yohanes Aris Purwanto, Heru Sukoco, Sony Hartono Wijaya

**Affiliations:** 1 Department of Computer Science, IPB University, Bogor 16680, Indonesia; 2 Department of Informatics, Faculty of Engineering, Krisnadwipayana University, Jakarta 13077, Indonesia; 3 Department of Mechanical and Biosystem Engineering, IPB University, Bogor 16680, Indonesia

**Keywords:** classification, fuzzy logic, mango, maturity, near infrared

## Abstract

Grading is a decisive step in the successful distribution of mangoes to customers according to their preferences for the maturity index. A non-destructive method using near-infrared spectroscopy has historically been used to predict the maturity of fruit. This research classifies the maturity indexes in five classes using a new approach involving classification modeling and the application of fuzzy logic and indirect classification by measuring four parameters: total acidity, soluble solids content, firmness, and starch. These four quantitative parameters provide guidelines for maturity indexes and consumer preferences. The development of portable devices uses a neo spectra micro development kit with specifications for the spectrum of 1350–2500 nm. In terms of computer technology, this study uses a Raspberry Pi and Python programming. To improve the accuracy performance, preprocessing is carried out using 12 spectral transformation operators. Next, these operators are collected and combined to achieve optimal performance. The performance of the classification model with direct and indirect approaches is then compared. Ultimately, classification of the direct approach with preprocessing using linear discriminant analysis offered an accuracy of 91.43%, and classification of the indirect approach using partial least squares with fuzzy logic had an accuracy of 95.7%.

## 1. Introduction

Mango has a fresh taste and distinctive aroma and contains various bioactive compounds such as vitamins, β-carotene, and polyphenols, which contribute to antioxidants and nutrients. Thus, mango has been recommended for daily consumption [1]. The mango has fairly large export potential, but the fruit also has perishable properties and a relatively short shelf life, so in distribution, there is often a decrease in quality and damage before the product reaches the customer [2]. Mango Arumanis can succeed in overseas markets because of its sweet, fresh, and slightly sour characteristics, which are flavors favored by many foreign consumers [3]. Arumanis mango’s characteristics are demanded by consumers from Korea, Japan, Singapore, Malaysia, Europe, and the Middle East. The existing advantages of this species make the selling price relatively higher than that of other types of local mangoes.

The determination of mango maturity at harvest is essential for distribution decisions in trade. A challenge for packinghouses in mango distribution is the grading stage, which is the initial stage that determines the success of the next level. Grading also determines decisions on sales, products whose shelf lives have been measured, and consumer preferences [4,5]. Grading aims to group mangoes with the same maturity level so that when the fruits arrive, they have a maturity level that is in accordance with consumer demand. Mangoes produce ethylene, which can trigger maturity in the surrounding mangoes [5]. Thus, the placement of mangoes with varying maturity levels in one box can cause the product to not meet consumer demands [3].

Arumanis mango is a climacteric fruit and usually harvested at the green stage. The fruit does not change its physical appearance or skin color during maturity, so it is difficult to determine the maturity levels of mangoes by sight compared to other types of mangoes that experience skin discoloration. Several grading methods are used in determining the maturity index based on the firmness, shape of the fruit, acidity, soluble solids content (SSC), and number of days after full bloom (DAF) [6,7]. Due to the subjective aspects of determining the mango maturity index based on physical characteristics, inconsistent assessments, and the use of destructive chemical parameters [8], a non-destructive method has been proposed as a tool to determine maturity. Near Infrared (NIR) spectroscopy is a non-destructive method that can be used to determine the maturity index of fruit [9,10]. NIR spectroscopy is a non-destructive analytical technique capable of providing chemical and structural information on certain samples in a very fast time [11]. NIR has a wavelength of 750–2500 nm. Using this technology, the target sample is illuminated with light, and the reflected light, or backscatter, is measured with a spectrometer. Compared to other infrared spectroscopy methods, NIR can increase the depth of penetration and has less stringent requirements for sample preparation [12,13]. The absorbance bands in the NIR region of the spectrum are often non-specific, broad, and overlapping. NIR spectrum analysis requires a multivariate method that is highly subject to noise arising from instrumentation, scattering effects, and measurement settings. Spectral transformation is an essential step in NIR processing because it can improve model performance [14]. The use of the best spectral transformation method is often determined through trial and error. Previously, a comparison of different spectral transformations was performed to achieve optimal model input [15].

In several previous studies, NIR spectroscopy was widely used to predict the chemical content and maturity of mangoes. However, the type of NIR spectroscopy used involves a desktop in the laboratory, which is quite large and expensive. Thus, this technology cannot be used for grading in the packinghouse. Although several studies to predict the chemical contents and parameters of mangoes using NIR spectroscopy have produced good accuracy, the results could still be improved. In a study to predict the total acidity (TA) content in mangoes, the predictive determination coefficient (R2) was found to be 0.89 using the standard normal variate (SNV) transformation and artificial neural networks [16]. A study on predicting firmness in Kent mangoes obtained an R2 of 0.75 using interval partial least squares (iPLS) [17].

Although there are commercially available portable NIR spectroscopy devices, including Scio [18], Neospectra [19], and F-750 [20], these devices are expensive and have not been able to classify fruits correctly; they also need to be calibrated for definite samples. The results of research using NIR spectroscopy still predict the content of starch, SSC, vitamin C, Firmness, TA, and dry matter in mangoes using a regression model, and no study has yet discussed the accuracy of the mango classification maturity index. Based on the recent literature, a portable NIR spectroscopy prototype was developed for the indirect classification of Chaunsa mango maturity based on dry matter content. This device was developed using a spectrometer model BIM-6002A with a spectrum of 400–1100 nm. The computing device was an Intel Compute Stick, and the development used Microsoft Visual C++ [21].

For classification of the maturity index, most studies in the literature recommend using an indirect approach based on the chemical content of the fruit. However, the comparison results of the direct classification model test are better than those of the indirect approach using the mango dry matter content threshold, with accuracy of 88.2% and 55.88% [21]. This paper proposes a new approach for classification of the mango maturity index indirectly using four parameters—TA, starch, firmness, and SSC—Using fuzzy logic. The selection of these parameters is based on the relevance between parameters and consumer preferences. There are currently no studies that use the parameters of TA, SSC, firmness, and starch simultaneously as a mango maturity index. The challenge in this study is the classification of five maturity indices due to each class being similar. We seek to improve the accuracy of the classification model by collecting and combining the methods and operations of spectral transformation, which include clipping, scatter correction, smoothing, derivation, trimming, and resampling methods, for further selection as the best model input.

## 2. Device Hardware and Software Development

**Figure 1:**
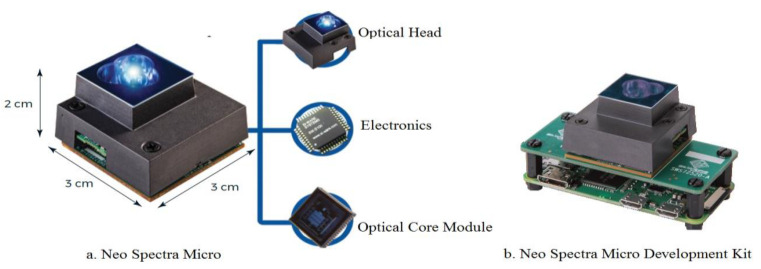
Portable NIR spectroscopy components (www.neospectra.com, accessed on 20 August 2022).

The portable device developed consists of a neo spectra micro development kit (NDK), touchscreen LCD, weight sensor, calibrator made of Barium Sulfate (BaSO4), power supply unit, and sample holder. The NDK consists of a Neo Spectra Micro (NSM), a Raspberry Pi board, and a software development kit. NSM is an integrated spectral sensor consisting of an optical head, electronics, and optical core module, with specifications for an NIR spectrum range of 1350–2500 nm, a signal-to-noise ratio of 2000:1, an integration time of up to 2 s, an optical resolution of 16 nm, and dimensions of 2 × 3 × 3 cm. The computing device is a Raspberry Pi equipped with a Broadcom BCM2835 processor and 512 MB LPDDR2 RAM. The components of the portable NIR device can be seen in Figure 1.

**Figure 2:**
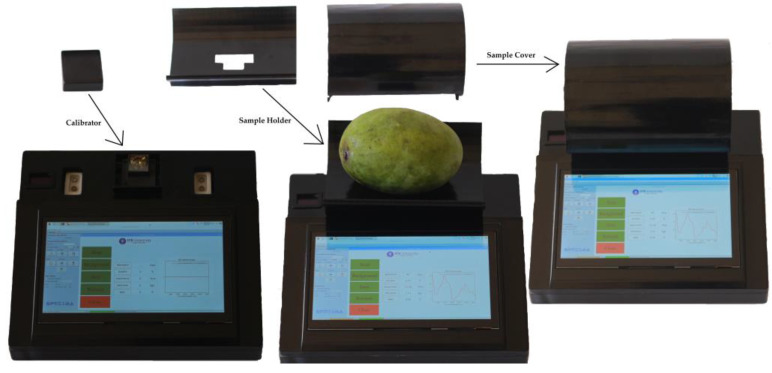
Portable NIR spectroscope.

One graphical user interface package used on portable devices consists of two pieces of software. Original software from the sensor manufacturer is used to control the spectrometer in parameter setting, calibration, and spectral data collection. The development software uses Python programming to obtain spectral information from samples; convert those data into a spectrum; remove noise; predict TA, SSC, firmness, and starch content; and classify maturity levels. The results of the manufacture and assembly of portable NIR devices can be seen in Figure 2.

## 3. Materials and Methods

### 3.1. Dataset

There is no standard for determining the maturity indexes of mangoes, but guidelines for the mango maturity index (Table 1) were issued by the Directorate General of Horticulture, Ministry of Agriculture of the Republic of Indonesia (2005). The maturity level is based on the age of the mango (i.e., the number of days after full bloom (DAF)), and the parameters of the color of flesh and taste are considered qualitative. The samples used in this study were Arumanis mango taken from the Situbondo plantation, East Java, and harvested at several levels of maturity: 80%, 85%, 90%, 95%, and 100%. Differences in the maturity index affect the quality, which includes the color of flesh, shelf life, and taste. The shelf life of mango decreases with an increase in sugar content and decrease in acid content.

**Figure 3:**
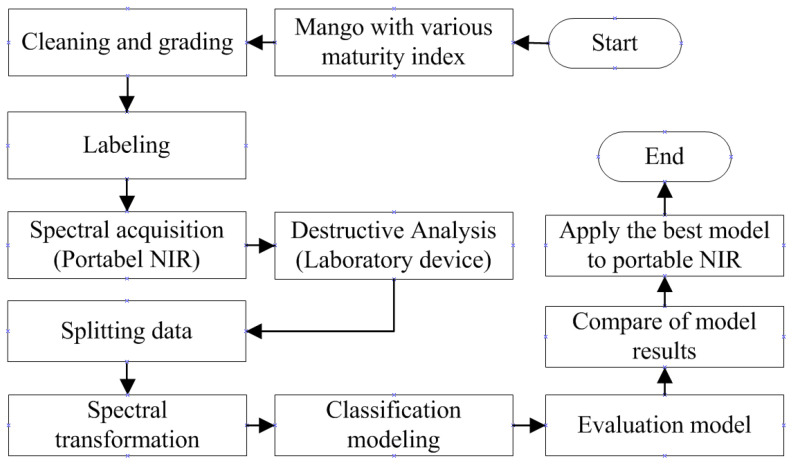
Research stages.

Samples were harvested on 23 September 2021. As many as 175 samples were taken, with a composition of 35 samples for each maturity index. Samples were harvested from the plantations and then cleaned, which included washing and sap removal, followed by the grading and packaging stages. The samples were then sent to the laboratory for labeling and non-destructive and destructive analysis. The stages of the research are shown in in Figure 3. Each sample was marked in as many as 4 locations and adjusted for the position of the mango when measuring on a portable NIR sensor, as shown in Figure 2. After obtaining spectral data and TA, SSC, firmness, and starch data at each location from all samples, then the data was split with a composition of testing data is 10%.

### 3.2. Spectral Acquisition

Before measuring the sample, the sensor was calibrated with the calibrator device followed by pressing the button on the application. After being calibrated, the sample holder containing the sample was placed and closed with a sample cover device; then, the scan button was pressed on the application. The device components are shown in Figure 2. For spectrum acquisition and reference analysis, four locations in each sample that correspond to the position of the NIR sensor at measurement were selected as representative of mango for TA, SSC, firmness, and starch content. The data stored on the device were then retrieved and processed for classification modeling.

Measurements of TA, SSC, firmness, and starch were performed using the same location as the measurement spectrum, with the firmness measurement stage first, followed by measurements of TA, SSC, and starch. For firmness measurements, a rheometer was used, while for TA and SSC measurements, an Atago pocket device with a sample depth of up to 40 mm was used after the skin was removed. For starch content, measurements were carried out only once for each sample. With a sample number of 175, we obtained a total of 700 spectral, TA, SSC, and firmness data respectively.

### 3.3. Spectral Transformation

Spectral transformation methods can improve model performance and interpretability [22]. These methods include clipping, scatter correction, smoothing, derivatives, trimming, and resampling. The order of preprocessing operations applied can affect the performance of the model [15]. Scatter correction was proposed to counteract the effect of particle size [22], while smoothing aims to smooth the NIR spectra and help eliminate environmental or instrumentation-related noise [23]. Clipping aims to remove or replace data points with values that exceed a user-defined threshold [24]. Trimming allows the extraction of continuous and non-continuous wavelength regions from full spectral data [25]. Resampling processes a new spectral resolution using the Fourier method that is able to combine the spectra obtained from several devices with different spectral resolutions [26]. Scatter correction consists of several operations: standard normal variate (SNV), multiplicative scatter correction (MSC), robust normal variate (RNV), localized version of SNV (LSNV), extended MSC (EMSC), normalization, and baseline. SNV can correct based on the mean and spectral standard deviation [27]. RNV is most suitable for data with significant noise, and the concept of correction is based on the median value and the interval between quartiles [28]. LSNV is conceptually similar to SNV with partial operations in the spectral window [27]. The MSC correction principle is only for the spectral mean, while the EMSC takes into account linear and quadratic correction [29]. Spectral normalization can be carried out over a certain range of values or using euclidean. The baseline only centers on the spectral mean. In this study, 5 methods and 12 operations were used with detailed operations, parameters, and values (Table 2).

### 3.4. Chemometrics

**Figure 4:**
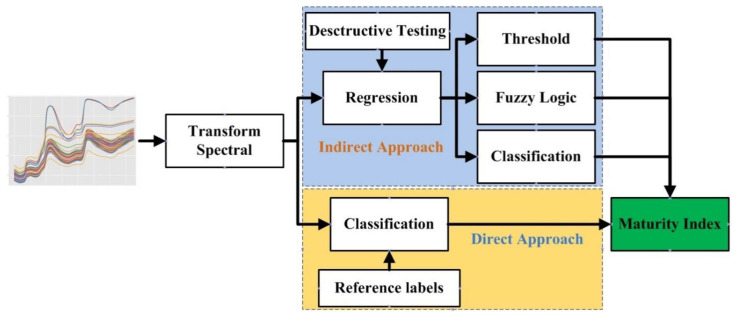
Approach for determining the maturity index.

In this study, we compare the accuracy of direct and indirect approaches for classifying the mango maturity index. The direct approach is to build a classification model using Support Vector Machine (SVM), K-Nearest Neighbor (KNN), Multi-layer Perceptron (MLP), Linear Discriminant Analysis (LDA), and Decision Tree (DT) based on the NIR spectrum and reference labels from the maturity index. Cross validation was carried out ten times with three repetitions and random sample selection. The indirect approach involves building a predictive model, first for the contents of TA, SSC, firmness, and starch. The regression model uses Partial Least Squares (PLS), SVM, Random Forest (RF), and Linear Regression (LR); the results are compared in terms of the correlation coefficient (R2) and root mean squared error (RMSE). The best regression model is then used for maturity index classification based on application of the threshold, fuzzy logic, and classification algorithms. Application of the threshold is based on statistical and expert calculations, and the classification algorithms compared are SVM, KNN, MLP, LDA, and DT. A schema of the mango maturity index classification with direct and indirect approaches is illustrated in Figure 4.

### 3.5. Fuzzy Logic

Fuzzy set theory is the basis for fuzzy logic, which is able to overcome subjective judgments [30]. The fuzzy set becomes the definition of membership functions and rules in linguistic data, which become a working model of fuzzy logic. The fuzzy set approach can overcome complex models that are not able to measure an indicator conventionally. Considering the reliability of operating on linguistic variables and a qualitative assessment system, fuzzy logic can work optimally in cases that cannot be described numerically. Fuzzy logic is able to solve ambiguities and inaccuracies in the problem of uncertainty [31] and was shown to be reliable in many fields of research, including predictions of peach quality based on acidity, SSC, and firmness parameters [32]; the determination of quality jute products based on 4 parameters [33], the selection of 6 varieties of pears with 10 criteria [34], sweet bell pepper maturity predictions in 4 classes [35], a prediction of hydrogen production from coffee mucilage and organic wastes using 5 parameters showing that fuzzy logic performance was better than an artificial neural network [36], and an evaluation of sensory quality in korla pear based on metabolites [37].

**Figure 5:**
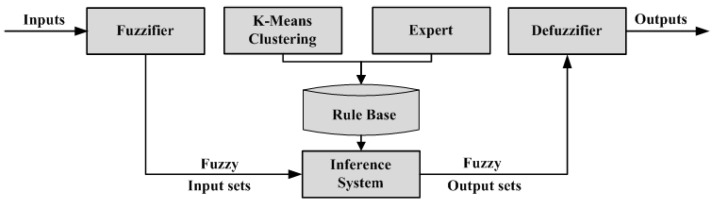
Diagram of a fuzzy inference system.

Fuzzy logic is the correct approach for determining the accuracy of mango maturity for several parameters. Processing input into output through a fuzzy inference system includes the membership function and if–then rule [38]. The formation of fuzzy sets by mapping data input points into fuzzy set membership values has an interval of 0–1. After the fuzzy set is formed, the minimum value of each method is sought based on the rules. The minimum curve value is aggregated to determine the maximum curve value [39]. From the maximum curve, the value is capable of being determined by defuzzification. Figure 5 shows the steps of the fuzzy inference system, namely, determining the input as a mango maturity parameter, making a fuzzy set and determining the degree of truth of each parameter, determining the inference rules in the system, and determining the output in the form of a fuzzy set that is calculated based on defuzzification. The trapezoidal membership function is chosen by considering the results of the destructive test. The membership functions of TA, SSC, firmness, and starch were built by considering the opinion of the expert assisted by the clustering approach using the K-means algorithm. The four parameters are grouped into high (H), medium (M), and low (L) categories. The output membership function is adjusted to the mango maturity index based on 5 levels, and a triangular membership function is selected for the output.

## 4. Results and Discussion

### 4.1. Destructive Test Results

The results of the destructive analysis for the content of TA, SSC, firmness, and starch are provided in Table 3. By measuring each mango sample taken at four locations selected to measure the chemical contents, 700 data were successfully obtained. However, four invalid spectral data were acquired, and destructive data at invalid locations were not used. Thus, the amount of data used was 696, with 139 data for each of the 80% and 95% maturity indexes, 140 data for each of the 85% and 90% maturity indexes, and 138 data for the 100% maturity index. A total of 70 data were ultimately used for testing.

Table 3 shows that a higher maturity index corresponds to a decrease in TA, starch, and firmness content and an increase in SSC content due to the conversion of starch to sugar. Based on the mean and SD values, the data are less varied and have a fairly high degree of closeness between maturity indices. The maturity indexes of 95% and 100% are very close in the SSC and starch parameters, the maturity indexes of 90% and 95% are very close for the TA parameter, and the maturity indexes of 80% and 85% are very close for the firmness parameter. Based on the expert and statistical analyses, thresholds were set for each maturity index for the content of TA, SSC, firmness, and starch, as shown in Table 4.

### 4.2. Non-Destructive Test Results

**Figure 6:**
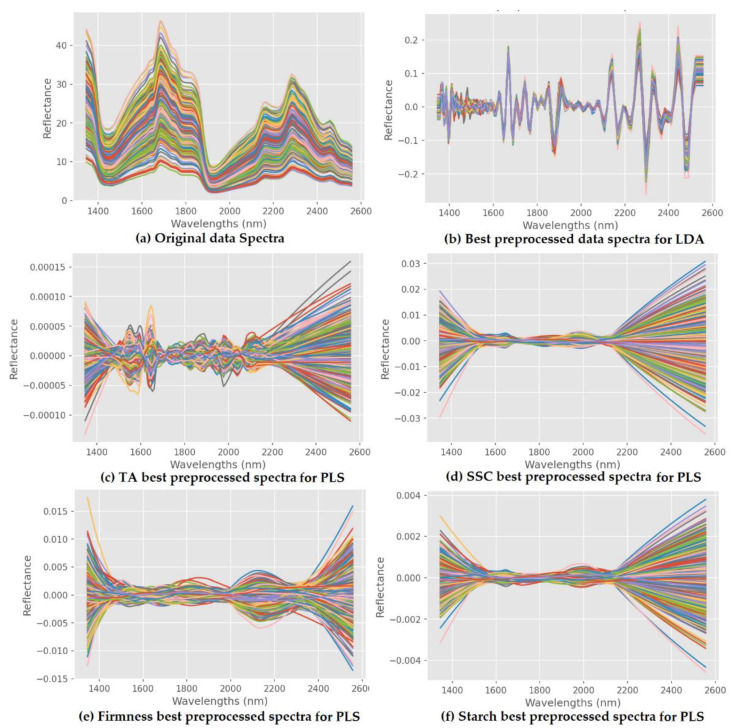
Original and spectral transformation of mango data spectra.

Figure 6a shows the data spectrum of the mango NIR, which was taken from a spectrum with a wavelength of 1350 to 2500 nm without spectral transformation. Here, each wave spectrum consists of 136 wavelength points with several spectral peaks and valleys. Figure 6b illustrates the results of the best spectral transformation in LDA classification, Figure 6c–f is the result of the best spectral transformation in PLS for the prediction of TA, SSC, firmness, and starch. The spectral transformation operations on each of the forming parameters can be seen in Table 5 and Table 6.

### 4.3. Direct Approach Model

This classification method is used to predict the mango maturity index. The maturity index labels include five indexes (Table 1). The predictor input includes 696 NIR spectra of Arumanis mango. The SVM, LDA, CNN, MLP, and DT classifiers are implemented using 10-fold cross-validation. Preprocessing is done via spectral transformation. Spectral transformation involves collecting and combining 12 spectral transformation operators from Table 2 with operations, parameters, and values for SAVGOL (filter_win = 5, 7, 11, 31, 71, poly_order = 3, deriv_order = 1, 2, 3) and SMOOTH (filter_win = 5, 7, 9, window_type = hamming). There are 1,152 operator combinations, and the operators that produce the best accuracy for SVM are CLIP {‘substitute’: None, ‘threshold’: 10,000.0}, MSC, RESAMPLE {‘resampling_ratio’: 0.7}, and SMOOTH {‘filter_win’: 5, ‘ window_type’: ‘hamming’}. The spectral transformation operator with the best accuracy for LDA is SAVGOL {‘deriv_order’: 3, ‘filter_win’: 5, ‘poly_order’: 3}. The spectral transformation operators with the best accuracy for kNN are CLIP {‘substitute’: None, ‘threshold’: 10,000.0}, DETREND {‘bp’: [0]}, RNV {‘in’: [75.0, 25.0]}, and SMOOTH {‘filter_win’: 5, ‘window_type’: ‘hamming’}. The spectral transformation operators with the best accuracy for MLP are CLIP {‘substitute’: None, ‘threshold’: 10,000.0}, DETREND {‘bp’: [0]}, MSC, RESAMPLE {‘resampling_ratio’: 0.7}, and SAVGOL {‘deriv_order ‘: 1, ‘filter_win’: 5, ‘poly_order’: 3}. The spectral transformation operators with the best accuracy for DT are CLIP {‘substitute’: None, ‘threshold’: 10,000.0}, DETREND {‘bp’: [0]}, MSC, and SAVGOL {‘deriv_order’: 3, ‘filter_win’: 11, ‘poly_order’: 3}.

After testing all classifiers without spectral transformation, LDA classifiers were found to have the best accuracy at 74.29%. After spectral transformation, all classifiers experienced an increase in accuracy, including an increase in SVM accuracy of 2.86%, an increase in LDA accuracy of 17.14%, an increase in kNN accuracy of 18.57%, an increase in MLP accuracy of 42.86%, and an increase in DT accuracy of 28.56%. Thus, the accuracy of LDA classifiers with spectral transformation increased to 91.43%. The results of the comparison of classifier accuracy can be seen in Table 5.

### 4.4. Indirect Approach Model

This section focuses on developing the optimal prediction model for TA, SSC, firmness, and starch by comparing SVM, PLS, RF, and LR regressors. Before modeling, preprocessing was carried out with spectral transformation, and the optimal number of components for PLS was determined. Spectral transformation involved the collection and combination of 12 spectral transformation operators from Table 2 with operations, parameters, and values for SAVGOL (filter_win = 7, 11, 21, 61, 121, poly_order = 3, 6, deriv_order = 0, 1, 2) and SMOOTH (filter_win = 7, 11, 61, window_type = hamming). There are 2112 operator combinations and operators that produce the smallest RMSE values for TA, namely, BASELINE, CLIP {‘substitute’: None, ‘threshold’: 10,000.0}, NORML, RESAMPLE { ‘resampling_ratio’: 0.7}, and SAVGOL {‘deriv_order’: 0, ‘filter_win’: 61, ‘poly_order’: 6}. The spectral transformation operators with the smallest RMSE values for SSC are CLIP {‘substitute’: None, ‘threshold’: 10,000.0}, DETREND {‘bp’: [0]}, NORML, RNV {‘in’: [75.0, 25.0]}, and SAVGOL {‘deriv_order’: 0, ‘filter_win’: 21, ‘poly_order’: 3}. The spectral transformation operators with the smallest RMSEs for firmness are CLIP {‘substitute’: None, ‘threshold’: 10,000.0}, DETREND {‘bp’: [0]}, EMSC, NORML, and SMOOTH {‘filter_win’: 11, ‘window_type’: ‘hamming’}. The spectral transformation operators with the smallest RMSEs for starch are CLIP {‘substitute’: None, ‘threshold’: 10,000.0}, DETREND {‘bp’: [0]}, EMSC, NORML, and RESAMPLE {‘resampling_ratio’: 0.7}. The results of the search for the number of components after running a loop that minimizes RMSE in PLS obtained the most optimal number of components: 86. The best model performance in predicting the content of TA, SSC, firmness, and starch in Arumanis mango was ultimately obtained with PLS. Comparative data are presented in Table 6. PLS offers the best performance for TA with R^2^ = 0.694 and RMSE = 0.155, for SSC with R^2^ = 0.920 and RMSE = 1.326, for firmness with R^2^ = 0.840 and RMSE = 0.387, and for starch with R^2^ = 0.931 and RMSE = 0.668.

Next, we use the predicted values for the content of TA, SSC, firmness, and starch from the best regression model to determine the mango maturity index based on the threshold in Table 4. Among the 70 test data, the TA accuracy is 55.71%. The maturity index of 80% successfully predicted all values correctly. The maturity index of 85% produced six incorrect predictions, and the maturity index of 90% only correctly predicted four values. The maturity index of 95% only correctly predicted three, and the maturity index of 100% incorrectly predicted 5. Here, the SSC accuracy is 71.43%, the firmness accuracy is 62.86%, and the starch accuracy is 68.57%. The results of the confusion matrix can be seen in Table 7.

The development of the classification model was compared using SVM, LDA, KNN, MLP, and DT. The training data used included 626 destructive test data for the content of TA, SSC, firmness, and starch, while the testing data were the result of predicting the contents of TA, SSC, firmness, and starch using PLS from 70 test data. The accuracy performance of each classification model is shown in Table 8.

### 4.5. Application of Fuzzy Logic

**Figure 7:**
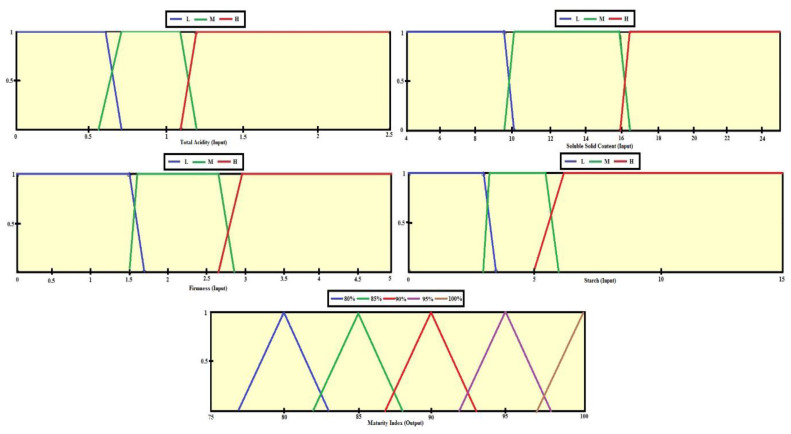
Membership function.

Mamdani is the fuzzy inference system used in this study. This method is very suitable because its foundation is experience or expert knowledge [30,40]. The four parameters in Table 3 are defined as inputs, namely, TA, SSC, firmness, and starch. The measurement results of the four parameters in numerical form are then used to build fuzzy logic membership functions in three classes: High (H), Medium (M), and Low (L). The determination of the level is based on the closeness of the values between the maturity classes in terms of parameters and consumer preferences. The trapezoidal membership function presents the ranking of the inputs, with numerical values of 0–2.5 for TA, 4–24 for SSC, 0–5 for firmness, and 0–15 for starch. The TA value is classified as L (0–0.65), M (0.6–0.8), and H (more than 0.75). SSC values are classified as L (0–9.6), M (9.2–16.5), and H (more than 16). Firmness values are classified as L (0–1.7), M (1.5–2.9), and H (more than 2.7). Meanwhile, starch values are classified as L (0–3.5), M (3–5.6), and H (more than 5.6). To obtain the numerical value of the maturity index, defuzzification is carried out. There are 5 values that can be seen in Figure 7, with a value range of 75–100. Based on the membership function of the input attribute, 81 inference rules are obtained. Table 9 presents the results of if–then rules obtained from expert opinions based on the processing of attribute clustering and expert analysis.

Using the same 70 test data in the classification and regression models, the predicted value for the four parameters was then determined via a fuzzy inference system process and obtained an accuracy result of 95.7%, with three incorrect prediction data that should be 95% guessed at 90% (Table 10).

### 4.6. Discussion

Mango maturity can be measured based on the total sugar content called SSC, which is the total sugar content that reflects the sweet taste of the fruit. Starch can also be used as a measure of fruit maturity. Starch and SSC have an inverse relationship: the more ripe the fruit is, the higher the SSC content and the more the starch content decreases due to the change from starch to sugar. The Arumanis mango’s sweet, fresh, and slightly sour characteristics, as well as consumer preferences for acidity and firmness levels, were chosen as the parameters for maturity. Acidity and firmness were also inversely proportional to SSC: the more ripe the mango, the lower the acidity and firmness levels. The maturity index labels used include five classes, 80%, 85%, 90%, 95%, and 100%, based on mango age from the Directorate General of Horticulture, Ministry of Agriculture of the Republic of Indonesia (Table 1). The direct classification approach uses class labels based on fruit age references, i.e., the number of days after the flower blooms. Determination of this fruit maturity class label can be applied to other fruits because it is easier to measure. Then, the class label can be linked to the chemical content of the fruit constituents.

The mango classifies maturity directly by training the spectra, which is compared to the class label. The resulting classification model identifies the test data according to which class label it belongs. Based on testing the maturity index classification directly, the LDA algorithm had higher accuracy than SVM, kNN, DT, and MLP. LDA offered 74.29% accuracy. However, by using SAVGOL {‘deriv_order’: 3, ‘filter_win’: 5, ‘poly_order’: 3} operations, the accuracy increased to 91.43%. In direct classification, the spectral transformation process increased the accuracy significantly, except for SVM.

The results in Table 5 and Table 6 showed that the spectral transformation affected and increased the accuracy of the model. In the spectral transformation, 12 spectral transformation operators with their parameters and values were collected and compared to achieve the best accuracy. Likewise, in the indirect classification approach, the spectral transformation process improved performance. In the indirect classification approach, predictions of the content of TA, SSC, firmness, and starch were carried out using a machine learning regression algorithm. The SVM, PLS, RF, and LR algorithms offered better performance than PLS, even though all of them had prediction errors. The next stage after prediction involves application of the threshold, classification modeling, and using fuzzy logic on the predicted value. The results of indirect classification produce the best performance through the application of Mamdani fuzzy logic with an accuracy 95.7% higher than that of the direct approach. The use of fuzzy logic can work well for boundary values between classes that are close to each other.

After scanning the mango with the NIR sensor on a portable device, the screen will display the maturity index value, TA value, SSC, firmness, and starch. The maturity index and values of the four parameters can be used as a reference for grading and product delivery. The higher the maturity index, the more suitable the product is for the nearest market.

## 5. Conclusions

In this study, we developed a portable NIR spectroscopy device using a neo spectra micro development kit with an NIR spectrum range of 1350–2500 nm and original software alongside Python programming to obtain spectral data, convert them into spectra, perform spectral transformations, and classify mango maturity indexes. In this way, a new approach to classify the mango maturity index, directly and indirectly, using fuzzy logic was presented. Maturity classification consists of five classes with measurements of four parameters, namely, TA, SSC, firmness, and starch. The spectral transformation process was carried out by collecting and combining 12 operators to obtain the best accuracy. The mango maturity index classification approach was conducted by comparing SVM, KNN, MLP, LDA, and DT. The best accuracy (91.43%) was obtained with the LDA and SAVGOL spectral transformation operations. The indirect approach begins with the spectral transformation process and compares SVM, PLS, RF, and LR. The best model performance in predicting the content of TA, SSC, firmness, and starch was PLS, with a total of 86 components. The classification accuracy using the threshold approach was SSC with 71.43%. The accuracy for starch was 68.57%, that of firmness was 62.86%, and that of TA was 55.71%. The classification accuracy with the four parameters using MLP was 85.71%, and the Mamdani fuzzy logic approach provided the best accuracy of 95.7%. When classifying the mango maturity index with five classes, where each class has proximity to the others, and the parameters used are correlated, the application of fuzzy logic increased the accuracy of the indirect approach, and the accuracy results were higher than those of the direct approach.

## Figures and Tables

**Table 1:** Arumanis mango maturity index.

Maturity Index	80%	85%	90%	95%	100%
Days after full bloom (DAF)	90–95	105	108	112	115
Color of flesh	Butter yellow around the seeds	Evenly butter yellow	Yellow orange	Orange	Reddish yellow
Taste	Sweet, sour, fresh	Sweet, sour, fresh	Sweet, fresh	Sweet, fresh	Sweet, fresh
shelf life (days)	21–25	14–17	7	5	1

**Table 2:** Methods, operations, and parameters of spectral transformation.

Method	Operation	Parameter	Value
Clipping	CLIP	threshold	Clipping
Scatter Correction	SNV		
	RNV	iqr	75–25, 90–10
	LSNV		
	MSC		
	EMSC		
	NORML		
	DETREND	bp	0
	BASELINE		
Smoothing	SMOOTH	filter_win	5, 7, 9
		window_type	hamming
Derivative	SAVGOL	filter_win	5, 7, 11, 31, 71
		poly_order	3
		deriv_order	1, 2
Resampling	RESAMPLE	rasio	0.7

**Table 3:** Summary statistics of TA, SSC, firmness, and starch for the Arumanis mango maturity index in 80, 85, 90, 95, and 100; n: amount of data; SD: standard deviation.

Maturity Index (%)	TA (%)				SSC (brix)				Firmness (kgf)				Starch (%)			
	Min	Max	Mean	SD	Min	Max	Mean	SD	Min	Max	Mean	SD	Min	Max	Mean	SD
80 (n = 139)	0.8	2.23	1.11	0.31	5.9	9.7	8.456	0.82	3.6	4.4	3.76	0.19	7.23	10.3	7.58	0.75
85 (n = 140)	0.53	0.81	0.67	0.11	9.5	15	11.26	1.16	3	3.6	3.47	0.09	5.3	7.19	6.52	0.65
90 (n = 140)	0.44	0.56	0.49	0.03	12.3	19.2	16.19	2.31	2.9	3.8	3.12	0.20	3.96	4.97	4.49	0.21
95 (n = 139)	0.36	0.46	0.42	0.03	18	19.7	19.15	0.51	1.8	2.9	2.13	0.32	2.01	3.26	2.31	0.45
100 (n = 138)	0.19	0.38	0.33	0.05	19.5	21.9	20.21	0.65	0.6	1.9	1.34	0.39	0.89	1.94	1.59	0.36

**Table 4:** Threshold of TA, SSC, firmness, and starch for Arumanis mango maturity index.

Maturity Index (%)	TA (%)	SSC (brix)	Firmness (kgf)	Starch (%)
80	>0.8	<9.5	>3.6	>7.2
85	0.55–0.8	9.5–13.5	3.4–3.6	5–7.2
90	0.45–0.55	13.5–19	2.9–3.4	3.5–5
95	0.37–0.45	19–19.6	1.8–2.9	1.95–3.5
100	<0.37	>19.6	<1.8	<1.95

**Table 5:** Comparison results of direct approach classification.

	Classification Testing				
	SVM	LDA	kNN	MLP	DT
NONE	58.57%	74.29%	32.86%	20.00%	22.86%
Transformation spectra	61.43%	91.43%	51.43%	62.86%	51.42%
Best operation	CLIP MSC RESAMPLE SMOOTH	SAVGOL	CLIP DETREND RNV SMOOTH	CLIP DETREND MSC RESAMPLE SAVGOL	CLIP DETREND MSC SAVGOL

**Table 6:** Results of indirect comparison of predictions.

		TA		SSC		Firmness		Starch	
		R^2^	RMSE	R^2^	RMSE	R^2^	RMSE	R^2^	RMSE
NONE	SVM	0.561	0.641	0.617	2.893	0.561	0.641	0.621	1.564
	PLS (86)	0.629	0.171	0.852	1.798	0.770	0.464	0.890	0.843
	RF	0.107	0.265	0.252	4.046	0.237	0.845	0.232	2.224
	LR	0.632	0.170	0.870	1.685	0.738	0.495	0.900	0.804
Transform	SVM	0.529	0.193	0.746	2.358	0.602	0.610	0.658	1.485
	PLS (86)	0.694	0.155	0.920	1.326	0.840	0.387	0.931	0.668
	RF	0.629	0.171	0.711	2.512	0.539	0.656	0.604	1.597
	LR	0.693	0.155	0.918	1.341	0.832	0.396	0.929	0.675
Best operation tranformation spectra		BASELINE CLIP NORML RESAMPLE SAVGOL		CLIP DETREND NORML RNV SAVGOL		CLIP DETREND EMSC NORML SMOOTH		CLIP DETREND EMSC NORML RESAMPLE SAVGOL	

**Table 7:** The results of the confusion matrix using PLS based on the threshold.

PLS		Predict																			
		TA					SSC					Firmness					Starch				
		80%	85%	90%	95%	100%	80%	85%	90%	95%	100%	80%	85%	90%	95%	100%	80%	85%	90%	95%	100%
Actual	80%	14					9	5				13	1				10	4			
	85%	6	10					16				6	4	6			5	11			
	90%		3	4	4	3		1	13					12	2			1	13		
	95%			8	3	2			5	3	5				4	9			1	6	6
	100%		1		4	8			3	1	9				2	11				5	8

**Table 8:** The results of the accuracy comparison of the classification approaches.

Algorithm	SVM	LDA	KNN	MLP	DT
Accuracy	80%	58, 57%	80%	85, 71%	68, 57%

**Table 9:** If–then rules for converting fuzzy inputs to fuzzy outputs.

	Firmness	H			M			L		
TA	SSC/Starch	H	M	L	H	M	L	H	M	L
H	H	80%	90%	95%	80%	90%	95%	80%	90%	95%
	M	80%	85%	95%	80%	85%	95%	80%	85%	95%
	L	80%	80%	95%	80%	85%	95%	80%	85%	95%
M	H	85%	90%	95%	85%	90%	95%	85%	95%	100%
	M	85%	85%	95%	85%	95%	95%	85%	85%	95%
	L	80%	85%	95%	80%	85%	95%	80%	85%	95%
L	H	85%	90%	95%	85%	95%	95%	85%	100%	100%
	M	85%	90%	95%	85%	90%	95%	85%	95%	95%
	L	80%	85%	95%	80%	85%	95%	80%	85%	100%

**Table 10:** The matrix results of testing using fuzzy logic.

Actual Classification		Fuzzy Algorithm Output				
		80%	85%	90%	95%	100%
80%	14	14				
85%	16		16			
90%	14			14		
95%	13			3	10	
100%	13					13

## Data Availability

The datasets that support the findings of this study are available in https://ipb.link/dataset-mangoes (accessed on 20 August 2022).
